# Informing a national rare disease registry strategy in Australia: a mixed methods study

**DOI:** 10.1186/s12913-023-10049-x

**Published:** 2023-10-31

**Authors:** Rasa Ruseckaite, Marisa Caruso, Chethana Mudunna, Falak Helwani, Nicole Millis, Susannah Ahern

**Affiliations:** 1 Department of Epidemiology and Preventive Medicine, Monash University, Melbourne, VIC Australia; 2 School of Public Health and Preventive Medicine, Monash University, Victoria, 3004 Australia; 3 Rare Voices Australia, Melbourne, VIC Australia

**Keywords:** Registry, Rare diseases, National strategy, Survey, Interviews

## Abstract

**Background:**

Rare disease registries (RDRs) facilitate monitoring of rare diseases by pooling small datasets to increase clinical and epidemiological knowledge of rare diseases and promote patient centred best practice. The aim of this study was to understand the current state of RDRs in Australia, data captured, impact on patient outcomes, funding models, and barriers and enablers regarding their establishment and maintenance.

**Methods:**

An exploratory sequential mixed methods study design was adopted. First, a list of Australian RDRs, primary contacts and data custodians was generated through online and consumer group (Rare Voices Australia (RVA)) contacts. A cross-sectional, anonymous online survey was distributed to registry custodians, managers, or principal investigators of 74 identified Australian RDRs, 88 RVA Partners, 17 pharmaceutical organizations and 12 RVA Scientific and Medical Advisory Committee members. Next, managers and coordinators of RDRs and databases who participated in the survey were invited to participate in semi-structured interviews. Quantitative and qualitative data were analysed using basic descriptive statistics and content analysis, respectively.

**Results:**

Forty RDRs responded to the survey; nine were national, five were based in Australia and New Zealand, and the remaining were global. Of the 40 survey respondents, eight were interviewed. Most of the RDRs captured similar information regarding patient characteristics, comorbidities and clinical features, diagnosis, family history, genetic testing, procedures or treatment types, response to treatments and complications of treatments. Better treatment outcomes, changes in process of care and changes in quality of care were the most frequently reported benefits of the RDRs. The main challenges proved to be cost/funding of data collection, data completeness, and patient consent. When asked, the participants identified opportunities and challenges regarding potential options to streamline RDRs in Australia in the future.

**Conclusion:**

Findings from this study highlighted significant dataset heterogeneity based on the individual disease, and current lack of interoperability and coordination between different existing RDRs in Australia. Nevertheless, a nationally coordinated approach to RDRs should be investigated given the particular benefits RDRs offer, such as access to research and the monitoring of new disease-modifying treatments.

**Supplementary Information:**

The online version contains supplementary material available at 10.1186/s12913-023-10049-x.

## Background

A rare disease (RD) is defined as one with a population prevalence of fewer than five in 10,000 people [1, 2]. While estimates of the number of RDs may vary between countries, due to varying definitions and challenges with data collection, it is prominently cited that there are more than 7,000 different RDs [3]. Approximately two million people in Australia live with a RD [4]. The impact of RD on a person’s life and their family can be devasting. Diagnosis can often take time because these conditions are complex. For many RDs, there are no effective treatments or cures.

Rare Voices Australia (RVA) in consultation with stakeholders across Australia developed the National Strategic Action Plan for Rare Diseases (the Action Plan) [5]. The Action Plan demonstrated the need for a national, coordinated and systematic approach to the collection and use of RD data, including registries. Clinical registries systematically monitor the quality of healthcare within specific clinical domains by routinely collecting, analysing and reporting health-related information [6]. Clinical registries have been developed for various health conditions and are powerful tools for reducing variation in clinical practice and improving healthcare quality, informing care management, research and health system planning [7].

In Australia, there are no national datasets for RDs, meaning that often estimates of the number and prevalence of RDs are based on international figures. There is also a lack of data on RD treatments and health outcomes. Without this data it is impossible to measure the overall burden and cost of RD in Australia. Registries can fill these data gaps and inform systems changes for better outcomes for people living with a RD [8]. Rare disease registries (RDRs) can enable recruitment to clinical trials. Patient reported outcome measures (PROMs) collected by some RDRs can inform a patient-centred approach to clinical care, by measuring meaningful outcomes for patients and their families [9].

Some RDs, such as cystic fibrosis [10], have benefited from long-standing Australian registries; however, standardised data collection sets or minimum data elements are not available for most disorders. RVA advocates for better health, disability and other systems for people living with a RD [4]. RVA is leading the collaborative implementation of the Action Plan, including the development of a national approach to person-centred RDRs to support national standards, best practice and minimum data sets [5].

A collaboration between the RVA Scientific and Medical Advisory Committee (SMAC) and Monash University researchers was established to understand the current landscape of RDRs both in Australia and internationally. A series of studies were conducted to describe the current state of the RDRs capturing Australian data. The first study was a scoping review of the literature in which we identified seventy-four RDRs [11]. The present study aims to explore data captured by the RDRs identified in the scoping review, to describe registry impact, barriers and enablers of data collection, registry funding, and the future potential of RDRs to improve patient outcomes, facilitate research and clinical trials and provide ongoing surveillance.

## Methods

### Mixed method design

In this study we adopted a mixed methods exploratory sequential study design [12]. A quantitative component using a cross-sectional survey was conducted first which was followed by qualitative interviews. Data were analysed separately and integrated at the final stage of the study in order to answer the research question.

### Survey

The survey was developed by Monash University researchers in collaboration with the RVA. It comprised four sections, with a total of 37 questions. The first section of the survey collected registry information, such as the number and type of RDs captured by RDRs, jurisdictions covered, data sharing, year established, participating sites, population of interest and size, ethics details, consent model, governance structure and funding source. The second section gathered information on RDR data collection, including parties responsible for data entry, methods of data collection, types and categories of data captured, outcomes collected, frequency and timing of data collection. The third section focused on research and reporting, and gathered information of stakeholders, frequency of data reporting, use of data and publication history. The final section collected qualitative data on the impact that registry has made, its challenges, barriers and enablers.

A list of Australian RDRs and RD organisations, contact details of their managers, investigators and data custodians was obtained through a search of online registry webpages and RVA contacts. The survey was distributed, via email, to registry custodians, managers, or principal investigators of 74 Australian RDRs, 88 RVA Partner organizations, 17 pharmaceutical organizations from RVA’s roundtable of companies and 12 RVA SMAC members.

The link to the survey included an invitation to participate, which explained the aims of the survey, its voluntary nature, and the requirements for participation. An implied consent process was utilised. A survey was administered online through Qualtrics Survey Software [13] from 19 to 2021 to 25 January 2022.

### Interviews

Registry custodians, managers, or principal investigators who responded to the survey were invited to participate in the second part of the study. We developed a semi-structured topic guide with open-ended questions (see Additional file 1). Interview topics covered objectives and research priorities for the RDRs, their functions in terms of improving health outcomes, resources required to maintain a registry, the coverage, attributes and feedback mechanisms to the different stakeholders such as clinicians, managers, policymakers and researchers, and barriers and enablers in achieving goals of the registry. Follow-up questions and prompts were used to obtain rich data. Critical discussions held throughout the analysis facilitated both self-reflection and common discussion for distinguishing between participant meaning and research interpretation.

Each potential participant who expressed their interest in the study was provided with an invitation letter, an explanatory statement and a consent form before the interview. All interviews were audio-recorded, subject to the participant’s consent. Consent was given verbally before the interview. It was not possible to establish how many participants saw the invitation to participate but decided not to volunteer. No participants dropped out of the research.

Interviews with the study participants were conducted on the phone by the experienced qualitative researcher (RR). On average, the interviews lasted 29 min (range 21–46). The interviews were conducted from 18 January to 12 February 2022.

### Data analysis

Quantitative data were statistically analysed in two stages using SPSS V26. Firstly, descriptive statistics were calculated for appropriate variables and responses were reported as both whole numbers and proportions. Secondly, sub-analyses by participant characteristics were undertaken for questions where the participant responses were varied.

The voice files of interviews were transcribed by a paid transcription service. To ensure data quality, the research team checked all transcriptions against the voice files. All participants were offered the opportunity to review transcripts. The process of analysing qualitative data involved coding and categorising the data from interview transcripts using NVivo software (Version 12, QSR, Australia). Transcripts were reviewed, and RR and MC thematically analysed the transcripts identifying quotes and words and grouping them according to themes and sub-themes as they emerged from the interviews [14]. Data saturation was determined when no new information was generated from successive interviews.

## Results

Managers and coordinators of 40 Australian RDRs responded to the survey (Table 1). Subsequently, the managers of these RDRs were invited to participate in the interviews. Eight people expressed their interest and were interviewed.

**Table 1:** Survey Results. Characteristics of Rare Disease Registries and Databases

Registry Name/Start/End Date	Patient/Clinician	Biobank	Jurisdiction/International Data Sharing	Patients/Episodes of Care (EOC), N Population captured, %	Consent	Governance	Funding
**Acrodysostosis Support and Research** 2022	Patient	No	AUS, UK, USA, Japan, China, South Africa, Italy, Sweden, Germany, Portugal	**Disease 1**: Acrodysostosis Type1 Patients - ~60 **Disease 2**: Acrodysostosis Type 2 Patients – 70 < 1%	Signed consent obtained for each episode of care	Steering/governing committee	Charity, Research grant
**Aplastic Anaemia and Other Bone Marrow Failure Syndromes Registry** 2013	Clinician	Yes: In progress, AUS bone marrow failure biobank	AUS No	Patients – 245; **Disease 1**: Aplastic Anaemia − 50%,**Disease 2**: Inherited BM -10%,	Signed consent not obtained but option to opt out	Steering/governing committee, Management group managing day-to-day activities, TOR exist for the SC	Charity, No explicit funds
**Australasian Interstitial Lung Disease Registry** 2016	Clinician	Blood, BAL, Tissue	ANZ No	NS Unknown	Signed consent obtained only on first contact with service	Steering/governing committee, Management group managing day-to-day activities, TOR exist for the SC	Private sector, Charity, CRE-PF
**Australian Autoinflammatory Disease Registry** 2015	Clinician	Yes: at WEHI	AUS No	Patients – 250 10%	Signed consent obtained only on first contact with service	Steering/governing committee, Management group managing day-to-day activities	Research grant
**Australian Bronchiectasis Registry** 2016	Clinician	No	AUS No	Patients – 1,700 1%	Signed consent not obtained but option to opt out	Steering/governing committee, TOR exist for the SC	Private sector, Research grant
**Australian Cystic Fibrosis Data Registry** 1998	Clinician	No	AUS No	Patients − 3,538 in 2020 90%	Opt in signed consent at first presentation & also opt out consent at some centres	Steering/governing committee, Management group managing day-to-day activities, TOR exist for the SC	Private sector, Charity
**Australian Genetic Heart Disease Registry** 2007	Clinician	No	AUS Yes	Patients − 2877 < 5%	Signed consent obtained only on first contact with service:	Steering/governing committee	No explicit funds
**Australian Haemoglobinopathy Registry** 2012	Clinician	No	AUS No	Patients − 765, EOC- 882 NS	Signed consent not obtained but option to opt out	Steering/governing committee, Management group managing day-to-day activities	Private sector
**Australian Idiopathic Pulmonary Fibrosis Registry** 2012	Clinician	Yes	AUS No	Patients − 867, EOC- 15,916 NS	Signed consent obtained only on first contact with service	Steering/governing committee, Management group managing day-to-day activities. TOR exist for the SC	Private sector, Research scholarship, Private philanthropic families
**Australian Leukodystrophy and White Matter Disorders Registry** 2020	Clinician	No	AUS No	Patients − 220 NS	Signed consent obtained only on first contact with service	Steering/governing committee	Public sector funding body (Dept. Human Services, Department of Health, university), Research grant
**Australian National Creutzfeldt-Jakob Disease Registry** 1993	Clinician	Yes: CSF, brain tissue	ANZ Ad hoc international collaborations with other prion disease surveillance centres or research units	Patients – 1,189 Close to 100%	Minimum data without consent under National Health Security Act 2007 (Cth). In depth case information with signed consent from next of kin	Management group managing day-to-day activities	Public sector funding body (e.g. Dept. of Human Services, Department of Health, university)
**Australian Neuromuscular Disease Registry** 2020	Clinician	No	AUS Yes, TREAT-NMD	NS NS	Consented to go on ANMDR, data updated annually with patient.	Steering/governing committee	Private sector, Charity, Reimbursed for deidentified data enquiries
**Australian Registry Network for Rare Lung Disease** 2009	Clinician	No	ANZ No	Patients – 85 30 rare lung diseases	Consent is not required as this registry has the authority to collect data via legislation.	Steering/governing committee	Charity
**Australian Rett Syndrome Database** 1993	NS	No	AUS No	Patients − 387 90%	Signed consent obtained only on first contact	Management group managing day-to-day activities	No explicit funds, Some funding from donations
**CHARGE Syndrome Australasia** 1994	Patient	No	ANZ No	NS 50%	NS	NS	NS
**Children with ILD research ANZ** 2016–2020	Clinician	No	AUS No	Patients – 36 90%	Signed consent obtained only on first contact with service	Steering/governing committee, Management group managing day-to-day activities	Research grant
**ConnectMPS** 2014	Patient/Industry	No	USA Yes	NS NS	Signed consent not obtained but option to opt out	NS	Private sector, Charity
**FOXG1 Patient Registry** 2015	Patient	Yes: FOXG1 Biobank at Corriel University	Many countries world wide The researchers can apply for access to the registration data	Patients – 412 50%	Signed consent obtained only on first contact with service	Management group managing day-to-day activities	Public sector funding body (e.g. Dept. of Human Services, Department of Health, university), Charity
**Global Angelman Syndrome Registry** 2019	Patient	Yes: Chromosome 15 Biobank	All countries Yes	Patients – 1,700 1%	e-consent on participation, can be changed at any time, or opt out	Steering/governing committee, Management group managing day-to-day activities, TOR exist for the SC	Charity
**Global Atypical Haemolytic Uremic SyndromeRegistry** 2012	Industry initiated	No	AUS, Belgium, Canada, Denmark, France, Germany, Israel, Italy, Poland&, South Korea, Spain, Taiwan, Turkey, UK, USA No	Patients – 1,976, EOC- 14,781 84%	Subjects sign consent at enrolment, then for any protocol amendments or special circumstances.	Steering/governing committee	Private sector
**Glomerular Diseases Registry & Biobank** 2020	Clinician	Yes: GRIT - housed at the NSW State-wide Biobank	AUS No	NS NS	Signed consent obtained only on first contact with service	Steering/governing committee, Management group managing day-to-day activities	Charity, Research grant
**International Fragile X Premutation Registry** 2020	Clinician, Patient	No	US, ANZ, India, some South American countries (intention is that it will be global) NS	NS < 10%	Signed consent obtained only on first contact with service	Steering/governing committee, TOR exist for the SC	Funded by USA university & USA Fragile X advocacy group registry initiated in the US
**International Pachyonychia Congenita Research Registry** 2004	Clinician, Patient	No	60 countries We share de-identified data with any credible researcher with a valid project	Patients - PC - Approximately 1500, 16 + in AUS NS	Signed consent obtained only on first contact with service	Steering/governing committee, Management group managing day-to-day activities	Charity
**Lysosomal acid lipase deficiency Registry** 2013	Industry initiated	No	AUS No	Patients − 0, EOC- 0 NS	N/A	Steering/governing committee	Private sector
**Lymphoma & Related Diseases Registry** 2016	Clinician	No	ANZ T-Cell Project 2.0, HoLISTIC Consortium, GLOW NLPHL Project, EU Research consortium on CLL Study	Patients − 4,656 NS	Signed consent not obtained but option to opt out	Steering/governing committee, Management group managing day-to-day activities, TOR exist for the SC	Private sector
**MGBase** 2021	Clinician	No	All within MSBase Yes, See MSBase	NS NS	Signed consent obtained only on first contact with service	Steering/governing committee, Management group managing day-to-day activities, TOR exist for the SC	Public sector funding body (e.g. Dept. of Human Services, Department of Health, university), Private sector, Charity
**Mito Registry** 2014	Patient	No	AUS No	Patients – 392 ~ 20%	Consent obtained during online registration, additional verbal consent over the phone during verification	Management group managing day-to-day activities	Charity
**Morquio A Registry Study** 2014–2024	Industry	No	82 sites across multiple countries NS	Patients − 325 in 2019 100%	Signed consent obtained only on first contact with service	Steering/governing committee, Management group managing day-to-day activities	Private sector
**Myeloma and Related Diseases Registry** 2012	Clinician	Yes: Myeloma 1000 biobank	ANZ No	Patients − 3415, Disease 2: Patients − 983, Disease 3: Patients – 375 ~ 25%	Signed consent not obtained but option to opt out	Steering/governing committee, Management group managing day-to-day activities	Private sector
**Neonatal Alloimmune Thrombocytopenia Registry** 2009	Clinician/Patient	No	AUS Yes, ethics in place	Patients-117, EOC- 134 ?60%	Signed consent not obtained but option to opt out	Steering/governing committee, Management group managing day-to-day activities, TOR exist for the SC	No explicit funds
**Observational, Longitudinal, Prospective, Long-Term Registry of Patients with Hypophosphatasia** 2014	Industry initiated	No	USA, UK, Germany, Japan, Spain, France, Italy, Australia, Canada, Israel, Austria, Saudi Arabia No	Patients – 986, worldwide 38 AUS 5–15%	Signed consent obtained only on first contact with service	Management group managing day-to-day activities	Private sector
**PFIC Advocacy & Resource Network Patient Registry** 2019	Patient	NS	Based in the USA but around 7 countries are registered Yes	NS NS	NS	Steering/governing committee, Management group managing day-to-day activities	Private sector, Research grant
**Poland Syndrome Community Register** 2021	Patient	No	No limit on countries, directory only in English NS	Patients − 150 NS	Signed consent obtained on first contact	Steering/governing committee	Charity
**Rare Genetic Lipid Disorder Registry** 2018	Clinician	No	WA No	Patients − 25 NS	Signed consent obtained only on first contact with service	Management group managing day-to-day activities	Public sector funding body (e.g. Dept. of Human Services, Department of Health, university), Research grant
**Sanofi Genzyme Rare Disease Registries** 1991- Gaucher Registry	Industry	No	Multinational across North America, Europe, Asia Pacific & Latin America No	Disease 1: Patients - Fabry: 7725 (AUS: 294), Disease 2: Patients - Gaucher: 6771 (AUS: 53), Disease 3: Patients - Pompe: 2835 (AUS: 18), Disease 4: Patients - MPS1: 1307 (AUS: 1)	Signed consent obtained only on first contact with service	Steering/governing committee, Management group managing day-to-day activities	Private sector
**Simons Searchlight** 2018	Clinician	Yes	USA No	Patients – 115 30%	Signed consent obtained for each episode of care	Management group managing day-to-day activities	Public sector funding body (e.g. Dept. of Human Services, Department of Health, university)
**The Australasian Myositis Registry** 2020	Clinician	No	AUS No	NS 5%	Signed consent obtained only on first contact with service	Management group managing day-to-day activities	Charity
**The Australian & New Zealand Vasculitis Quality & Disease Registry** 2022	Clinician	No	ANZ No	NS 10%	Signed consent obtained only on first contact with service	Steering/governing committee, Management group managing day-to-day activities, TOR exist for the SC	Private sector, Charity
**Thrombotic Microangiopathy Registry** 2009	Clinician	No	ANZ No	Patients − 138 NS	Signed consent not obtained but option to opt out	Steering/governing committee, Management group managing day-to-day activities, TOR exist for the SC	No explicit funds

*ACFDR: Australian Cystic Fibrosis Registry, aHUS: Atypical Hemolytic Uremic Syndrome, AIPFR: Australian Idiopathic Pulmonary Fibrosis Registry, ANCJDR: Australian National Creutzfeldt-Jakob Disease Registry, ANMDR: Australian Neuromuscular Disease Registry, ANZ: Australia & New Zealand, ARNOLD: Australian Registry Network for Rare Lung Disease, AUS: Australia, BAL: Bronchoalveolar lavage, BM: Bone Marrow, CLL: Chronic Lymphatic Leukaemia, ConnectMPS: Connect Mucopolysaccharide, CRE-PF: Centre Research Excellence, Pulmonary Fibrosis, CSF: Cerebrospinal fluid, Cth: Commonwealth, Dept: Department, DNA: Deoxyribonucleic acid EOC: Episodes of Care, ERIC: European Research Initiate on Chronic Lymphatic Leukaemia, FOXG1: Forkhead Box G1, GLOW: Global Nodular lymphocyte Predominant Hodgkin Lymphoma One Working Group, GOSH: Great Ormond Street Hospital, GRIT: Glomerular Diseases Registry & Biobank, HbR: Haemoglobinopathy, HoLISTIC: Hodgkin Lymphoma International Study for Individual Care, ILD: Interstitial Lung Disease, LAL-D: Lysosomal acid lipase deficiency, LARDR: Lymphoma & Related Diseases Registry, MGBase: Myasthenia Gravis Base; MRDR: Myeloma & Related Diseases Registry, MSBase: Multiple Sclerosis Base, NAIT: Neonatal Alloimmune Thrombocytopenia, NLPHL: Nodular lymphocyte Predominant Hodgkin Lymphoma, NS: Not stated, NSW: New South Wales, NT: Northern Territory, PC: Pachyonychia Congenita, PFIC: Progressive Familial Intrahepatic Cholestasis, SC: Steering Committee, TOR:Terms Of Reference, TREAT-NMD*: Treat *Neuro Muscular Disease, UK: United Kingdom, US: United States of America, WA: Western Australia, WEHI: Walter & Eliza Hall Institute*

### Survey results

#### General registry characteristics

Eighteen (45%) registries recorded data relating to a single RD. The remaining registries collected data from numerous RDs or conditions (e.g. the Australian Mitochondrial Disease Foundation Patient Registry collects data from more than 50 different RDs). Ten (25%) RDRs provided biobank facilities. Eleven (28%) RDRs were patient-initiated.

The number of patients in each registry varied, with the mean (SD) of 1249 (1826) reported by 28 (70%) RDRs. For example, the Rare Genetic Lipid Disorder Registry recorded data from 25 patients; however, the Sanofi Genzyme Rare Disease Registries captured data from 7,725 patients with Fabry disease. The Morquio A Registry Study stated that their population coverage was nearly 100% of Australian patients who were under the Life Saving Drugs Program. The Australian National Creutzfeldt-Jakob Disease Registry (ANCJDR) reported similar coverage. Thirty-five (87.5%) registries required a consent and ethics approval. Twenty-two (55%) RDRs were funded primarily via the public or private sector, a charity (18, 45%) or via a research grant (9, 23%). Most registries (29, 73%) were managed by a steering/governing committee or a management group (27, 68%), with established terms of reference.

#### Data collection methods

In most registries (34, 85%), data were entered online by clinicians or staff members (Table 2), but there were a few exceptions.

**Table 2:** Survey Results. Data collected by Rare Disease Registries and Databases

Registry Name	Data Input	Data Collection Method	Demographic Information	Diagnostic/Clinical Variables	Treatment/Procedure Information	Adverse Events Information	Quality of Life and Other Outcomes	Time Points
**Acrodysostosis Support & Research**	Patient/Carer, Clinician	Online/Webpage	RDs: Acrodysostosis Type1, Acrodysostosis Type 2 Given name, Surname, DOB, Gender, Country of birth	Age at diagnosis, Year of diagnosis, Diagnostics tests, Clinical visits, Pregnancy/childbirth, Comorbidities, Family history of disease, Genetic variations, Other	Clinical management, Imaging, Therapy, Laboratory results, Medications/frequency/dosages, Lifestyle intervention, Surgical procedures	Medical device, Cause investigation, Health effects, Complications	Survival/mortality	Initial diagnosis/procedure; 12mths, 24mths
**Aplastic Anaemia and Other Bone Marrow Failure Syndromes Registry**	Clinician, Registry Staff	Direct from EMR (electronic medical record/heath record)	Given name, Surname, DOB, Gender, Country of birth	Age at diagnosis, Year of diagnosis, Time taken to diagnosis, Diagnostics tests, Clinical visits, Comorbidities, Family history of disease, Genetic variations	Clinical management, Therapy, Laboratory results, Medications/frequency/dosages Treatments, Procedures	NS	Survival/mortality	NS
**Australasian Interstitial Lung Disease Registry**	Clinician	Online/Webpage	Given name, Surname, DOB, Gender, Postal address, Email address, Ethnicity	Age at diagnosis, Year of diagnosis, Diagnostics tests, Clinical visits, Comorbidities, Family history of disease	Clinical management, Imaging, Laboratory results, Medications/frequency/dosages, Supplemental Oxygen, Lung Tx, Lung Bx Hospitalisation	Health effects, Chest/Cardiovascular/Extremities/Joints/Skin/Lymphatics/Acute Exacerbations	Survival/mortality	Initial diagnosis/procedure (6mths, 12mths, 24mths, other). Baseline, then ongoing 6 monthly visits
**Australian Autoinflammatory Disease Registry**	Registry Staff	Telephone call	Given name, Surname, DOB, Gender, Postal address, Email address	Age at diagnosis, Diagnostics tests, Family history of disease, Genetic variations	Clinical management, Therapy, Laboratory results	NS	NS	At single-time point at Recruitment
**Australian Bronchiectasis Registry**	Clinician	Online/Webpage	Given name, Surname, DOB, Gender, Postal address, Email address, Country of birth	Age at diagnosis, Diagnostics tests, Clinical visits, Comorbidities, Genetic variations	Clinical management, Imaging, Laboratory results, Medications/frequency/dosages	NS	PROMs/PREMs: QoL-B, BHQ	Initial diagnosis/procedure (6mths, 12mths, 24mths, other)
**Australian Cystic Fibrosis Data Registry**	Clinician, Other: Nursing staff	Direct from EMR (electronic medical record/heath record), Online/Webpage, Electronic upload submission/template spreadsheet	DOB, Gender, Country of birth, the first 2 initials of the given name & surname first 2 initials	Age at diagnosis, Year of diagnosis, Time taken to diagnosis, Diagnostics tests, Clinical visits, Pregnancy/childbirth, Comorbidities, Genetic variations, anthropometry	Clinical management, Imaging, Therapy, Laboratory results, Medications/frequency/dosages, Lifestyle intervention, Surgical procedures	Medical device, Cause investigation, Health effects	NS	Initial diagnosis/procedure (6mths, 12mths, 24mths, other); quarterly data for remainder of time person seen at the clinic.
**Australian Genetic Heart Disease Registry**	Registry Staff	Direct from EMR (electronic medical record/heath record), Telephone call, Postal/Paper, Electronic upload submission/template spreadsheet	Given name, Surname, DOB, Gender, Postal address, Email address, Country of birth	Age at diagnosis, Year of diagnosis, Diagnostics tests, Clinical visits, Comorbidities, Family history of disease, Genetic variations	Clinical management, Imaging, Therapy, Laboratory results, Medications/frequency/dosages Surgical procedures	Medical device	Survival/mortality	Registry will aim to update data records on an annual basis.
**Australian Haemoglobinopathy Registry**	Clinician, Registry Staff	Online/Webpage	Given name, Surname, DOB, Gender, Country of birth	Year of diagnosis, Diagnostics tests, Comorbidities, Family history of disease, Genetic variations	Clinical management, Imaging, Laboratory results, Medications/frequency/dosages, Chelation treatment, Transfusion therapy	Complications related to haemoglobinopathies	NS	On multiple occasions
**Australian Idiopathic Pulmonary Fibrosis Registry**	Registry Staff	Postal/Paper, Other: Collection of Clinical results from clinicians entered into the database. Collection of HRCT scans Collection of histopathology glass slides.	Given name, Surname, DOB, Gender, Postal address, Email address, Ethnicity	Age at diagnosis, Year of diagnosis, Diagnostics tests, Clinical visits, Comorbidities, Family history of disease	Clinical management, Imaging, Therapy, Laboratory results, Medications/frequency/dosages Surgical procedures, Radiotherapy	NS	Survival/mortality PROMs/PREMs: Shortness of Breath, Cough & Wheeze, Gastro-Oesphageal Reflux, St George Respiratory Questionnaire HADS, Sleepiness & tiredness.	On multiple occasions: Initial diagnosis/procedure (6mths, 12mths, 24mths, other). For AIPFR Stage 1 participants data is captured every 6-months. For AIPFR Stage 2 participants the protocol is for collection of data & samples at Baseline, at 3 months & 6 months from baseline & then every 6 months from 12 months from Baseline.
**Australian Leukodystrophy & White Matter Disorders Registry**	Registry Staff	Direct from EMR (electronic medical record/heath record), Online/Webpage, Telephone call	Given name, Surname, DOB, Gender, Postal address, Email address, Country of birth	Age at diagnosis, Year of diagnosis, Diagnostics tests, Family history of disease, Genetic variations	NS	NS	Survival/mortality	Initial diagnosis/procedure (6mths, 12mths, 24mths, other)
**Australian National Creutzfeldt-Jakob Disease Registry**	Registry Staff	Other: Diagnosing clinician & public health units under National Health Security Act 2007 (Cth), hospital records with consent from NOK	Given name, Surname, DOB, Gender, Postal address, Country of birth	Age at diagnosis, Year of diagnosis, Diagnostics tests, Comorbidities, Family history of disease, Genetic variations, time to death, strain typing, risk factors diagnostic tests, disease progression, epidemiological risk factors	NS	NS	Survival/mortality	diagnosis to death
**Australian Neuromuscular Disease Registry**	Registry Staff	Direct from EMR (electronic medical record/heath record), Telephone call	**RDs: SMA, FSHD, DMD/Becker, Myotonic Dystrophy**: Given name, Surname, DOB, Gender, Postal address, Email address, Country of birth	Year of diagnosis, Diagnostics tests, Clinical visits, Comorbidities, Family history of disease.	Clinical management, therapy, Laboratory results, Medications/frequency/dosages	Health effects	PROMs/PREMs	6mths, 12mths
**Australian Registry Network for Rare Lung Disease**	Clinician	Online/Webpage	DOB, Patient initials, postcode, medical record number	NS	NS	NS	NS	Monthly
**Australian Rett Syndrome Database**	Researchers request parent-reported data	Online/Webpage, Telephone call	Given name, Surname, DOB, Gender, Postal address, Email address, Country of birth	Age at diagnosis, Diagnostics tests, Pregnancy/childbirth, Comorbidities, Genetic variations	Clinical management, Therapy Surgical procedures	NS	Survival/mortality, PREMS caregiver reported	Every 3–4 years approximately
**Children with ILD Research Australia & New Zealand**	Clinician, Registry Staff	Postal/Paper	Given name, Surname, DOB, Gender, Postal address, Country of birth	Age at diagnosis, Year of diagnosis, Diagnostics tests, Comorbidities, Family history of disease, Genetic variations	Clinical management, Imaging, Therapy, Laboratory results, Medications/frequency/dosages	NS	Survival/mortality	At enrolment
**Connect MPS**	Patient/Carer	Online/Webpage	Given name, Surname, DOB, Gender, Postal address, Email address, Country of birth	Age at diagnosis, Year of diagnosis, Time taken to diagnosis, Diagnostics tests, Clinical visits, Genetic variations	Clinical management, Therapy, Laboratory results, Medications/frequency/dosages, Lifestyle intervention	NS	PROMs/PREMs	On multiple occasions
**FOXG1 Patient Registry**	Patient/Carer	Online/Webpage	Given name, Surname, DOB, Gender, Postal address, Email address, Country of birth	Age at diagnosis, Year of diagnosis, Diagnostics tests, Clinical visits, Genetic variations	Clinical management, Imaging, Therapy, Laboratory results, Medications/frequency/dosages, Lifestyle intervention Surgical procedures	NS	Growth post PEG surgery, seizure changes	6mths- As the parents update it
**Global Angelman Syndrome Registry**	Patient/Carer	Online/Webpage	Given name, Surname, DOB, Gender, Postal address, Email address, Country of birth	Age at diagnosis, Year of diagnosis, Time taken to diagnosis, Diagnostics tests, Clinical visits, Pregnancy/childbirth, Comorbidities, Genetic variations	Clinical management, therapy, Laboratory results, Medications/frequency/dosages, Lifestyle intervention Surgical procedures	Effects of medications, Complications	PROMs/PREMs,	On multiple occasions, Initial diagnosis/procedure (6mths, 12mths, 24mths, other)
**Global Atypical Hemolytic Uremic Syndrome Registry**	Patient/Carer	Online/Webpage	Given name, Surname, DOB, Gender, Postal address, Email address, Country of birth	Age at diagnosis, Year of diagnosis, Time taken to diagnosis, Diagnostics tests, Clinical visits, Pregnancy/childbirth, Comorbidities, Genetic variations	Clinical management, therapy, Laboratory results, Medications/frequency/dosages, Lifestyle intervention	Effects of medications Adverse Events, Complications	PROMs/PREMs,	NS
**Glomerular Diseases Registry & Biobank**	Clinician	Direct from EMR (electronic medical record/heath record)	Given name, Surname, DOB, Gender	Age at diagnosis, Year of diagnosis, Diagnostics tests, Comorbidities, Genetic variations	Clinical management, Laboratory results, Medications/frequency/dosages, Lifestyle intervention	NS	Survival/mortality	Initial diagnosis/procedure (6mths, 12mths, 24mths, other)
**International Fragile X Premutation Registry**	Patient/Carer	online survey form completed by patient or their agent	Given name, Surname, DOB, Gender, Postal address, Email address, Occupation, Education, Marital status, Employment status, Household income, Race/Ethnicity, Religion	Age at diagnosis, Year of diagnosis, Time taken to diagnosis, Diagnostics tests, Family history of disease, Genetic variations, Height, Weight, Neurological problems, Autoimmune problems, Fertility/menstruation questions, Age of menopause, Other specific health issues listed (hypertension, diabetes, kidney disease etc.)	NS	NS	PROMs/PREMs	Not sure if registrants are required to update their information?
**International Pachyonychia Congenita Research Registry**	Patient/Carer, Clinician, Registry Staff	Online/Webpage	Given name, Surname, DOB, Gender, Postal address, Email address, Country of birth	Age at diagnosis, Diagnostics tests, Family history of disease, Genetic variations	Clinical management, Imaging, Laboratory results, Medications/frequency/dosages, Lifestyle intervention	Health effects	PROMs/PREMs	patients are able to update their data as needed
**Lysosomal acid lipase deficiency Registry**	Registry Staff	Direct from EMR (electronic medical record/heath record)	DOB, Gender	Age at diagnosis, Diagnostics tests, Clinical visits, Pregnancy/child’s birth, Family history of disease, Genetic variations	Imaging, Therapy, Laboratory results	Imaging, Dosing & patient safety Adverse Events	NS	NS
**Lymphoma and Related Diseases Registry**	Clinician, Other: Study Coordinators on site	Online/Webpage	Given name, Surname, DOB, Gender, Country of birth	Age at diagnosis, Year of diagnosis, Time taken to diagnosis, Diagnostics tests, Clinical visits, Pregnancy/childbirth, Comorbidities, Family history of disease, Genetic variations	Clinical management, Imaging, Therapy, Laboratory results, Medications/frequency/dosages Surgical procedures, Radiotherapy, Chemotherapy treatment	Health effects, Complications	Survival/mortality	Initial diagnosis/procedure (6mths, 12mths, 24mths, other). Any other review beyond 24 months when patient has a status change
**MGBase**	Clinician	Online/Webpage	Given name, Surname, DOB, Gender	Age at diagnosis, Year of diagnosis, Diagnostics tests, Clinical visits, Pregnancy/childbirth, Comorbidities, Family history of disease, Genetic variations, outcome scales	Surgical procedures	Health effects, hospitalisation, Complications	Survival/mortality PROMs/PREMs	On multiple occasions, Initial diagnosis/procedure (6mths, 12mths, 24mths, other)
**Mito Registry**	Patient/Carer, Registry Staff	Online/Webpage, Telephone call	Given name, Surname, DOB, Gender, Postal address, Email address	NS	NS	NS	NS	When person with mito registers, with regular verification as information changes (6–24 months)
**Morquio A Registry Study**	Clinician	Online/Webpage	DOB, Gender	Age at diagnosis, Year of diagnosis, Diagnostics tests, Clinical visits, Pregnancy/childbirth, Comorbidities, Genetic variations	Clinical management, Imaging, Therapy, Laboratory results, Medications/frequency/dosages Surgical procedures	Health effects, complications	Survival/mortality	Initial diagnosis/procedure (6mths, 12mths, 24mths, other); 6 monthly visits or as required
**Myeloma and Related Diseases Registry**	Clinician, Registry Staff	Online/Webpage	Given name, Surname, DOB, Gender	Age at diagnosis, Year of diagnosis, Time taken to diagnosis, Diagnostics tests, Comorbidities, Family history of disease	Clinical management, Imaging, Therapy, Laboratory results, Medications/frequency/dosages Surgical procedures, Radiotherapy, Chemotherapy	NS	Survival/mortality	On multiple occasions, Initial diagnosis/procedure (6mths, 12mths, 24mths, other)
**Neonatal Alloimmune Thrombocytopenia Registry**	Clinician, Registry Staff	Online/Webpage	Given name, Surname, DOB, Gender	Age at diagnosis, Year of diagnosis, Diagnostics tests, Pregnancy/childbirth, Family history of disease	Clinical management, therapy, Laboratory results, Medications/frequency/dosages, Transfusion of blood products	Clinical outcomes for mother & baby, Complications	Survival/mortality	usually post-delivery
**Observational, Longitudinal Prospective, Long-Term Registry of Patients with Hypophosphatasia**	Clinician	Direct from EMR (electronic medical record/heath record), Telephone call	DOB, Gender	Age at diagnosis, Year of diagnosis, Time taken to diagnosis, Diagnostics tests, Clinical visits, Pregnancy/childbirth, Comorbidities, Family history of disease, Genetic variations	Clinical management, Therapy, Laboratory results, Medications/frequency/dosages	Cause investigation, Adverse Events	Survival/mortality, PROMs/PREMs: age appropriate	NS
**PFIC Advocacy and Resource Network Patient Registry**	Patient/Carer	Online/Webpage, Postal/Paper	Given name, Surname, DOB, Gender, Postal address, Email address, Country of birth	Age at diagnosis, Time taken to diagnosis, Diagnostics tests, Pregnancy/childbirth, Comorbidities, Genetic variations	Clinical management, Laboratory results, Medications/frequency/dosages Surgical procedures	Complications	PROMs/PREMs,	can add information at any point
**Poland Syndrome Community Register**	Patient/Carer	Online/Webpage	Given name, Surname, DOB, Gender, Postal address, Email address, Country of birth	Age at diagnosis, Year of diagnosis, Time taken to diagnosis, Diagnostics tests, Clinical visits, Pregnancy/childbirth, Comorbidities, Family history of disease, Genetic variations	Lifestyle intervention	NS	PROMs/PREMs	6mths
**Rare Genetic Lipid Disorder Registry**	Clinician, Registry Staff	Direct from EMR (electronic medical record/heath record), Other: LIS	Given name, Surname, DOB, Gender, Email address, Country of birth	Age at diagnosis, Year of diagnosis, Time taken to diagnosis, Diagnostics tests, Clinical visits, Pregnancy/childbirth, Comorbidities, Family history of disease, Genetic variations	Clinical management, Imaging, Therapy, Laboratory results, Medications/frequency/dosages, Lifestyle intervention Surgical procedures	Complications	Survival/mortality	Initial diagnosis/procedure (6mths, 12mths, 24mths, other); At follow-up clinic appointments
**Sanofi Genzyme Rare Disease Registries**	Registry Staff	Direct from EMR (electronic medical record/heath record)	**RDs: Fabry. Gaucher, Pompe, MPS1** DOB, Gender, ethnicity	Age at diagnosis, Year of diagnosis, Diagnostics tests, Clinical visits, Pregnancy/childbirth, Genetic variations, immunogenicity	Clinical management, Imaging, Therapy, Laboratory results, Medications/frequency/dosages, Other (audiology, PFT’s, biopsy, stress tests, ECG, ECHO, cardiac events, cerebrovascular events)	Complications	Survival/mortality , PROMs/PREMs	On multiple occasions, Initial diagnosis/procedure (6mths, 12mths, 24mths, other) - it is a prospective & retrospective longitudinal disease registry. All available data is collected, usually once or twice a year according to patient clinical management schedule
**Simons Searchlight**	Patient/Carer	Online/Webpage, Telephone call, Electronic upload submission/template spreadsheet	Given name, Surname, DOB, Gender, Postal address, Email address, Country of birth	Age at diagnosis, Year of diagnosis, Time taken to diagnosis, Diagnostics tests, Clinical visits, Pregnancy/childbirth, Comorbidities, Family history of disease, Genetic variations	NS	NS	Survival/mortality	12mths
**The Australasian Myositis Registry**	Clinician, Registry Staff	Online/Webpage	DOB, Gender	Age at diagnosis, Year of diagnosis, Diagnostics tests, Clinical visits, Comorbidities, Family history of disease, Genetic variations	Clinical management, Imaging, Therapy, Laboratory results, Medications/frequency/dosages, Lifestyle intervention Surgical procedures, Radiotherapy, Chemotherapy	Health effects	Survival/mortality	Recruitment visit
**The Australian and New Zealand Vasculitis Quality & Disease Registry**	Patient/Carer, Clinician, Registry Staff	Online/Webpage, Postal/Paper	**RDS: ANCA-associated vasculitis, Giant Cell Arteritis** Given name, Surname, DOB, Gender, Postal address, Email address, Country of birth	Age at diagnosis, Year of diagnosis, Time taken to diagnosis, Diagnostics tests, Clinical visits, Comorbidities, Family history of disease, Family history of disease	Clinical management, therapy, Laboratory results, Medications/frequency/dosages	Health effects	PROMs/PREMs: AAV-PRO, EQ-5D-5 L	Initial diagnosis/procedure (6mths, 12mths, 24mths, other)
**Thrombotic Microangiopathy Registry**	Clinician	Online/Webpage	Given name, Surname, DOB, Gender, height, weight	Year of diagnosis, Diagnostics tests, Comorbidities	Clinical management, Therapy, Laboratory results, Medications/frequency/dosages Surgical procedures, plasma exchange	Cause investigation, Health effects	NS	6mths, 12mths, 24mths, 18 months, 2.5 years, 3 years

*AV-PRO:ANCA-associated Vasculitis Patient-Reported Outcome Questionnaire, ACFDR: Australian Cystic Fibrosis Data Registry, aHUS: Atypical Hemolytic Uremic Syndrome, AILDR: Australasian Interstitial Lung Disease Registry ; AIPFR: Australian Idiopathic Pulmonary Fibrosis Registry, ANCA: Antineutrophil Cytoplasmic Antibody, ANMDR: Australian Neuromuscular Disease Registry, ANCJDR: Australian National Creutzfeldt-Jakob Disease Registry, ANMDR: Australian Neuromuscular Disease Registry, ARNOLD: Australian Registry Network for Rare Lung Disease, BHQ: Bronchial Hyperresponsiveness Questionnaire, Bx: Biopsy, ConnectMPS: Connect Mucopolysaccharide, Cth: Commonwealth, DMD: Duchenne muscular dystrophy, DOB: Date of Birth, ECG: Electrocardiogram, ECHO: Echocardiogram, EMR: Electronic Medical Record, EQ-5D-5 L European Quality of Life Five Dimensional Questionnaire, FOXG1: Forkhead Box G1, FSHD: Facioscapulohumeral Muscular Dystrophy, HADS: Hospital Anxiety and Depression Scale, HbR: Haemoglobinopathy, HRCT: High-resolution computed tomography, ILD: Interstitial Lung Disease, LARDR: Lymphoma & Related Diseases Registry, LAL-D: Lysosomal acid lipase deficiency, LIS: Laboratory Information Systems, MGBase: Myasthenia Gravis Base, Mito: Mitochondrial Disease, MPS1: Mucopolysaccharidosis I, NAIT: Neonatal Alloimmune Thrombocytopenia, NOK: Nest Of Kin, NS: Not Stated, PEG: Percutaneous Endoscopic Gastrostomy, PFIC: Progressive Familial Intrahepatic Cholestasis, PFT: Pulmonary Function Test, PREMs: Patient Reported Experience Measures, PROMs: Patient Reported Outcome Measures, QoL-B: Quality of Life – Bronchiectasis, RD: Rare Disease, SMA: Spinal Muscular Atrophy, TMA: Thrombotic Microangiopathies, Tx: Transplant*

These included the Australian Autoinflammatory Disease Registry collecting data through telephone calls only, and the Australian Idiopathic Pulmonary Fibrosis Registry (AIPFR) and Children with Interstitial Lung Disease Research Australia and New Zealand collected data via postal/paper methods. Five (12.5%) registries collected data via electronic medical record.

#### Demographic information

Thirty-eight (95%) registries answered the survey questions regarding type of data collected by their registry. Of these, thirty-seven (97%) registries captured patient name, surname, date of birth, gender, postal and email address and country of birth. Additionally, some registries (5, 13%) captured information about ethnicity, race, religion, occupation, education, marital status, employment status and income.

#### Diagnostic/clinical variables

Thirty-seven registries (92.5%) collected patient diagnostic data. These included age and year of diagnosis, diagnostic tests, clinical visits, pregnancy/childbirth, comorbidities, family history of disease and genetic variations. Some registries captured additional diagnostic and clinical data specific to their registries. For example, Sanofi Genzyme RDRs collected details of immunogenicity, and the International Fragile X Premutation Registry records neurological, autoimmune problems, fertility/menstruation information, age of menopause, as well as information on other specific health issues such as hypertension, diabetes and kidney disease.

#### Treatment/Procedure and adverse events data

Clinical assessment and treatment data were captured by thirty-two registries (80%). This information included any/all of clinical management, imaging, therapy, laboratory results, medication type, frequency of treatment dosages and lifestyle interventions.

Information on procedure types (e.g. surgical procedures, radiotherapy and chemotherapy) was entered by twenty-six (65%) RDRs. In addition, the Australasian Interstitial Lung Disease Registry collected information on lung transplants and lung biopsies; the Australian Haemoglobinopathy Registry (HbR) and the Neonatal Alloimmune Thrombocytopenia (NAIT) Registry both captured transfusion data. The Sanofi Genzyme RDR recorded information on audiology, pulmonary function tests, biopsies, stress tests, ECG, cardiac events and cerebrovascular events; and the Thrombotic Microangiopathy Registry collected data on plasma exchange.

Only twenty-one registries (52.5%) captured data of adverse events and complications. This information included details of medical devices, cause investigations, adverse health effects, and clinical outcomes for mother and baby.

#### Other outcomes and quality of life data

Nineteen (47.5%) RDRs reported patient survival or mortality. Further to this, sixteen registries (40%) collected PROMs or Patient Reported Experience Measures. The Australian Bronchiectasis Registry uses the Quality of Life-Bronchiectasis Questionnaire and the Bronchiectasis Health Questionnaire. The Australian Idiopathic Pulmonary Fibrosis Registry collected shortness of breath, cough and wheeze, gastro-oesophageal reflux, St George Respiratory Questionnaire, hospital anxiety and depression, and sleepiness and tiredness instruments. The Australian and New Zealand Vasculitis Quality and Disease Registry used the ANCA-associated Vasculitis Patient-Reported Outcome Questionnaire and the EQ-5D-5 L. The Global Atypical Haemolytic Uremic Syndrome (aHUS) Registry utilised the FACIT Fatigue Scale v4 (Adults), Paediatric FACIT and the Patient Questionnaire (Adults and Paediatrics) to collect quality of life data.

#### Timing of data collection

Six registries (15%) captured data at a single-time point. Thirty-two registries (80%) collected data on multiple occasions, including at initial diagnosis, at time of procedure, or at 6, 12- and 24-month intervals. Some registries (5, 13%) collected their data more regularly. Specifically, the Australian Cystic Fibrosis Data Registry (ACFDR) collected data at 3-month intervals, and the ANCJDR and the Observational Longitudinal Prospective Long-Term Registry of Patients with Hypophosphatasia collected data at each clinic visit.

#### Output reporting and data use

Most registries (33, 83%) reported their outputs to multiple stakeholders including clinicians, hospitals/sites, jurisdictions, consumers, funders, industry and government departments. For example, the Australian HbR reported its output only to their funders. The ANCJDR and the Global aHUS Registry reports output to government departments only, and the Lysosomal acid lipase deficiency (LAL-D) Registry reported their output to regulatory agencies only.

Eight (20%) registries published annual reports. Two (5%) RDRs reported their data quarterly (e.g. AILDR and Simons Searchlight) or twice a year (AIPFR and Myeloma and Related Diseases Registry). Six registries (15%) have not produced any reports.

#### Data use for research purposes/post-marketing surveillance

Thirty-three registries (82.4%) used their data for research and publication purposes, including clinical trials, epidemiological modelling, collaborative projects, secondary use of data and availability of registries for data access requests.

Nine registries (22.5%) used their data for post-marketing surveillance for high-cost medicines. These registries included the ACFDR, Australian HbR, AIPFR, Australian Neuromuscular Disease Registry, MGBase, Morquio A Registry Study, aHUS Registry, LAL-D Registry, and the Observational, Longitudinal, Prospective, Long-Term Registry of Patients with Hypophosphatasia.

#### Registry impact on patient outcomes

Registry managers and coordinators were asked to describe the impact they have made on patient outcomes. The distribution of responses and exemplary quotes are shown in Table 3. The top three responses included “*better treatment outcomes*”, “*changes in process of care*” and “*changes in quality of care*”.

**Table 3:** Survey Results. Summary of Impact of Rare Disease Registries and Databases on Patients Outcomes, Barriers and Enablers in Meeting Registry objectives

Category	N (%) of respondents *	Exemplary quotes
**Impact on patient outcomes**		
Change in process of care	9 (22.5)	*“[Allows] understanding of disease and ability to communicate to physicians” (RDR30)*
Quality of care	9 (22.5)	*“A wide range of research has increased understanding of a rare and fatal disease with limited treatment options and a prognosis similar to Lung Cancer. Linked education and support services have increased health care professional and consumer understanding of this disease” (RDR7)* *“It gives hope that new treatments and better understanding of the disease will follow” (RDR21)*
Treatment outcomes	9 (22.5)	*“Understanding of natural history and treatment variations” (RDR20)*
Other	6 (15.0)	*“[…] assisted researchers in addressing many of the unanswered questions surrounding the clinical course and outcomes” (RDR33)* *“understanding of disease and ability to communicate to physicians” (RDR30)* *“supported reimbursed access in Australia” (RDR18)*
**Enablers**		
Patient participation	23 (57.5)	*“The decision to focus on a small data set to support participation in research studies has enabled this registry to succeed” (RDR1)*
Funding	20 (50.0)	*Please see* **Barriers**
Expertise	20 (50.0)	*“A committed and collaborative Steering Committee of Principal Investigators lead by an enthusiastic and energetic individual who as Chair has tirelessly driven the project, sought funding and facilitated research has been instrumental in the success of the registry” (RDR7)*
Consent	16 (40.0)	NA^#^
Volunteers	11 (27.5)	*“Grateful for the registry and the patient’s associations in particular the [registry] for supporting [registry] within the broader […] foundation” (RDR17)*
Identified data	6 (15.0)	*“We have a treasure chest of 12 years’ worth of data and would love to collaborate with researchers to look at long-term outcomes for our babies.” (RDR20)*
**Barriers**		
Funding	21 (52.5)	*“Sustainability the main problem” (RDR11)* *“This registry was established using common data elements from the equivalent USA and EU ones- sadly due to lack of funds it is now nascent. We need an Australian RD registry which we can use!!” (RDR12)* *“We would also love to have some base funding to ensure the registry is kept alive and active, especially as new treatments are being developed. […] Lack of understanding about registries from governance offices, lack of funding, lack of awareness of condition” (RDR20)*
Availability of data	13 (32.5)	*“Any way that we can get a registry or more data for Australian patients we are desperately keen to do so. Showing a presence/need in Australia is vital for Australian patients accessing lifesaving medications.” (RDR21)* *“Availability of data due to low frequency of rare disease populations” (RDR6)*
Patient participation	12 (30)	*“[Lack of] understanding of the value of being part of the registry while clinical trials for mitochondrial disease are still limited” (RDR1)* *“Australian patients have also noted that they have signed up, but never heard anything more.” (RDR13)* *“[Lack of] Diversity in population” (RDR33)*
Staff levels	11 (27.5)	*“Motivation by clinicians” (RDR18)*
Volunteers	6 (15.0)	NA
Identified data	6 (15.0)	*“transparency of data use” (RDR13)*
Consent	5 (12.5)	*“governance issues across states take up a lot of registry staff’s time” (RDR16)* *“Interstate ethical approval processes” (RDR29)*
**Challenges**		
Data completeness issues	28 (70.0)	*“The parents are so worn out from the stress of having a […] child that it can be overwhelming to continually update the registry” (RDR31)* *“Patients need to tick a box to allow data to be used in reports some people simply miss this box or don’t tick” (RDR21)* *“Australian patients not accustomed to/familiar with registries” (RDR34)*
Cost of data collection	16 (40.0)	*“[Lack of] resources when unfunded” (RDR28)* *“No funding for case entry” (RDR20)*
Consent issues	11 (27.5)	*“Significant delays have been caused by the different ethical and governance required by each state and then by individual health districts. Managing the online ethics portals for each state is also burdensome!!” (RDR40)*
Data validity	8 (20.0)	*“This database is going through a process of streamlining to implement a minimum dataset, improve the existing database and bring all contributing sites under one protocol” (RDR4)* *“Rare condition–so clinicians unaware and potential cases may be missed” (RDR20)* *“complexity of information that could be captured regarding the large variety of diseases” (RDR1)*
Time allocated to data entry	4 (10.0)	*“Time required” (RDR17)* *“lack of clinician time to enter cases” (RDR20)*
Data security	4 (10.0)	*“Little transparency on content or use of data” (RDR13)*

* Total N = 40

*# no quote available*

Six (15%) registries indicated “*other*” type of impacts made on patient outcomes. For example, the RDR33 “*assisted researchers in addressing many of the unanswered questions surrounding the clinical course and outcomes*.” The RDR30 increased the “*understanding of disease and ability to communicate to physicians*” while the RDR18 “*supported reimbursed access in Australia*”.

#### Enablers, challenges and barriers towards operating RDRs

Patient participation (57.5%) was recognised as the main enabler towards operating RDRs followed by registry funding (50%), expertise in running a RDR (50%), consent of participants (43%), volunteer participation (30%) and identified data collection (16%).

52.5% of registries listed funding as the main barrier towards successful maintenance of the RDR, followed by the availability of data “*due to the low frequency of people living with particular RDs*” (32.5%), patient participation (30%), low registry staffing levels (27.5%), issues around identifiable data (15%), a lack of volunteers (15%) and consent issues (12.5%).

Other challengers included data completeness, reported by 28 (70%) of respondents and “*cost/funding of data collection*,” mentioned by 16 (40%) of participants. Eleven (27.5%) of survey participants listed consent issues, 8 (20%) mentioned data validity while the remaining participants stated data security and time allocated to data entry as major concerns for their registries (Table 3).

### Interview results

Four interview participants were volunteers who worked with clinicians across the country and internationally in their organisations. Other participants had a professional role before taking up the role with their registry. Most of the participants came into the RD area because they either had a family member or friend with a RD. Four of the participating interviewees belonged to global registries with the size of their RDRs ranging from 80 to 500 patients.

Table 4 displays the discussion topics and sample quotes provided by the interview participants. The topics included “*Data collected*”, “*Registry platforms*”, “*Registry Management and Consumer Involvement*”, “*Post-marketing Surveillance*”, “*Funding*”, “*Registry Impact*”, “*Challenges*” and “*Views towards RVA’s National Strategy*”.

**Table 4:** Interview results. Themes and exemplary quotes with the participants of Rare Disease Registries and Databases

Topic	Sample quotes
Data collected	*“[…] basic details of patients and families and donors” and only “occasionally, we get dates of birth but […], often just information on donations and fundraising” (RDR42)* *“It’s annual, but to be honest, in reality it’s not happening annually” (RDR40)*
Registry platform	*“Using REDCap to collect the data” (RDR44)* *“We’ve got a CRM in place now[.] so all of the information is in one database” (RDR41)* *“[…] using FileMaker Pro […] and it’s a relational database […]. We keep that on a secure server, so it’s difficult – one of our biggest challenges has been other sites getting access to that database, and we’ve never really worked out a good way to do it” (RDR33)*
Registry Management and Consumer Involvement	*“We put together a steering committee, [.] newly-appointed medical advisory panel […] other invested clinicians […] a consumer rep” (RDR43).* *“One person from each of those sites is on the steering committee. And then all major decisions would go through the steering committee” (RDR33)* *“The stakeholders are the community and the organisations and experts have been involved. So, the registry was created by a group of organisations and researchers and medical professionals who have had a long-term interest in treatments (RDR34)* *“[…] we do have a steering committee. Which is fairly large [….], it’s 18 committee members” (RDR40)*
Post-marketing Surveillance	*“[…] really not in a position to give them (pharmaceutical companies) that data” (RDR43).* *“[…] a lot of the clinical trials that they run are generally international clinical trials, because they don’t have the patient numbers for them to realistically be viable in Australia” (RDR42)*
Funding	*“We’ve received some small grants through […] external philanthropy trusts. But the idea is that once our fundraiser gets up and running, that we will pitch it to pharma and other organisations that perhaps have an interest in some of the data that might be produced” (RDR43)* *“We have a relationship with Pfizer, […] sponsored small amounts of our fundraising events” (RDR42)* *“[…] it’s relied on – essentially not much funding ever, so a lot of goodwill from investigators and a lot of patching together money from other grants” (RDR33)* *“We’re getting more funding now from […]. we’ve been able to make a bit more headway and at the moment we’re trying to convert, which is a big task” (RDR40)* *“[…] we fundraise for all of our activities” (RDR1)*
Registry impact	*“No publication to date. Because the data is still quite new and there is such a small sample size, […] it takes a very long time to start seeing trends, to start identifying our trends through these registers. So, nothing has been published to date” (RDR44)* *“I’m not sure that the registry has made any direct impact yet. It’s quite new still, in the sense that it’s still being rolled out to various countries” (RDR34)* *“…we’ve used the registry so far to recruit, to tell people about clinical trials and other research projects, but we have not measured whether telling people about those projects lead to them being recruited” (RDR1)*
Challenges	***Funding***: *“Funding is obviously one of the big ones” (RDR43)* *“[…] sustainable funding is a challenge. We do have three third party funders that if we lost them, we would be in trouble because we don’t get any government funding, any direct government funding” (RDR41)* *“[…] our main focus is fundraising, […] getting other people aware of our disease and invested in helping us fundraise to keep funding research, I think is probably our main focus” (RDR42)* *“[…] we do not have the recurrent funding to participate in the costs or to fund the costs of our involvement in an existing registry. So, capacity, size, funding are huge barriers” (RDR34)* ***Resources***: *“Resourcing, getting everybody on the same page. And the information to be able to do all of that is not readily available. So, there is not a one-stop shop that says ‘This is how you get up a national register” (RDR44)* *“[…] getting the resources where they’re needed, but finding out what is needed” (RDR41)* *“[…] We’re so limited in being able to recruit the participants that we want, being able to get the data entered in the way that we want. In so many ways, we’re limited because we don’t have the resources” (RDR33)* *“There’s a lot of data that we can’t collect on our clients and our community members because we’re not a provider and so we can’t collect health information” (RDR43)* *“[…] more than 55% of our audience is in regional, rural locations. So, actually getting to those people […] and collecting biological samples […] and then storing then, again, (is) extremely hard” (RDR44)* ***Ethics and database***: *“We’ve been reluctant to push sites to complete data knowing that, […] things are going to change slightly because we’ve put under NMA and this is the new approved database that we want you to use, so it’s not at its” (RDR40)*
Views towards RVA’s National Strategy	***Purpose***: *“What are they trying to include in that registry? Is it a registry of registries? Because all the data that would be collected for different conditions would be quite different in many circumstances” (RDR43)* *“We should be focussed on what the real goal is, which is being able to combine data to get an accurate snapshot or accurate information about all the [strains] of rare diseases, and let’s keep an open mind about what the solution looks like” (RDR1)* ***Benefits of collaboration between RVA and RD groups***: *“[…] shared knowledge about running registries could be really useful. The better job that we can do, […] the more that the patients will benefit. […] collectively amongst us, we’ve got […] expertise and solutions that we need and different ways of doing things […]. Feeling like we’re connected to a community of people trying to run the rare disease registries will be really useful” (RDR33)* *“Need the leaders to help us all and guide us all and drive us all” (RDR41)* *“It would be good if we had a united voice […]. But there’s so many rare diseases aren’t there, and some are more prevalent than others” (RDR41)* *“[…] the integration component would be really beneficial for the national register. […] that way you’ve got a good pool of data straight off the bat as opposed to having to start from scratch” (RDR44)* *“Rare Voices is very, very good at working with organisations and collaborating and coming up with a common platform and that clearly would be important because you would want t […] be in a position where you could bring new conditions to the table” (RDR34)* ***Concerns***: *“So conditions which don’t have registry you could encourage them to participate in the registry but I’m guessing part of the challenge would be having organisations that already have registries converting across to a new platform” (RDR34)* *“Rare Voice is all over this but it’s about tapping into individuals, in particular, families at the various points at which they’re receiving information about a condition or the condition’s treatments. […] that would be kind of a multilayered approach to informing people about the registries and inviting participation” (RDR34).* *“Being able to facilitate bringing all that information together confidentially would be a big challenge. Having just seen how difficult getting patients to put their details on the global patient register for their own disease has been, I’m not sure how you would go about collecting that information” (RDR42)* *“Having worked inside of tiny, under-resourced patient organisations, and having worked with clinicians that are often trying to help in their minimal spare time, with research projects, I find it really challenging because this national versus international tension is difficult” (RDR1)* *“The criteria for success are that data can be pooled with international data, and I think if we had one Australian registry, I just don’t see how that can happen without […] patients and clinicians having to contribute to multiple (databases)” (RDR1)* *“[…] when I think about the idea of having a national rare disease registry, or standard, is that (it) allows us to have data that is across multiple rare diseases, how does that fit in for a disease where you do want to be able to join international research?” (RDR1)* *“[…] the only other question is with the RVA register, are they looking at using REDCap or are they looking at using a different platform? I’m looking at it from a tech perspective and a logistics project management perspective” (RDR44)* *“Standardised guidelines, to me, absolutely. And standardisation in terms of integration, perhaps, as well. One of the discussions that we’ve had within the clinician team was people wanting it to integrate with neuromuscular registries but not necessarily doing so or whether it’s appropriate to do so and things like that as well. It’s something that the registry community would need to be fully involved with and invested in, for RVA to do this” (RDR43)* *“[…] it’s really about more flexible than the idea of a single registry in Australia, I think we need to look at data standards and […] ongoing funding for joining up of data in order to have regular reporting of rare diseases in Australia, rather than aiming for everyone to be part of one set of infrastructures” (RDR1)*

In terms of the data collected, registry management, consumer involvement, funding and impact made, the interviews confirmed findings from the survey.

When asked on the impact made, the interview participants said that impact was either not measured or no direct impact has been made. This appeared due to either a small sample size of the registry participants or a slow roll out of the registry.

When asked regarding the challenges faced when maintaining a registry, the respondents agreed that funding was the most pressing issue. Other challenges included fundraising activities, resources required to setup and maintain a registry, ethics and governance, and database issues.

The participants were also asked to provide their feedback regarding the RVA’s national strategy around centralised data set, and what that would it look like with a national registry of RDs. Main comments included feedback regarding the goals and priorities: “*We should be focussed on what the real goal is, which is being able to combine data to get an accurate snapshot or accurate information about all the [strains] of rare diseases, and let’s keep an open mind about what the solution looks like*” (RDR1). Other responses included benefits of collaboration between the RVA and RD groups: “*shared knowledge about running registries could be really useful. The better job that we can do, […] the more that the patients will benefit. […] Feeling like we’re connected to a community of people trying to run the rare disease registries will be really useful*” (RDR33).

Comments around challenges of a national registry included concerns for existing registries, education and participation for patients, confidentiality, and on what level would a national registry engage with international RDRs. Participants were cautious regarding the possible impact of the national registry: “*Being able to facilitate bringing all that information together confidentially would be a big challenge. Having just seen how difficult getting patients to put their details on the global patient register for their own disease has been, I’m not sure how you would go about collecting that information*” (RDR42).

Other comments raised on the discussion around support for a national registry included partnering with international registries, platform, standardised guidelines and challenges around funding: “*The criteria for success is that data can be pooled with international data, and I think if we had one Australian registry, I just don’t see how that can happen without […] patients and clinicians having to contribute to multiple (databases)*” (RDR1).

## Discussion

To our knowledge, this was the first mixed-methods study of RDRs’ capturing Australian data and investigating the impact of these RDRs on patient outcomes, data captured, funding sources, governance, and the barriers and enablers of building and managing these registries. RDRs collecting Australian data vary in their purpose, coverage, treatment types, target population, data collected, governance models and sources of funding. Information collected by most RDRs include patient demographics, diagnostic and laboratory data, as well as data related to therapies and treatments. Common challenges to data collection reported in the survey and interviews were data completeness, errors and other data quality issues. To prevent such errors, harmonization of the data items, as well as identification and correction of causes of poor data quality should be continuously considered [15–17]. Using standard coding and classification systems along with the standard definitions for data items should also be considered to enhance comparability [18]. To improve the quality of registry data, RDR managers should develop clear definitions for core data elements, to ensure that procedures for checking the data are specified, and that feedback has been provided [15].

Numbers of patients captured by RDRs and quality of data collected may relate to the impact of registries on patient outcomes. The quality of a registry is closely associated with the validity of information that is published through reports and other forms of dissemination [19]. Thus, publications from registries have great value in guiding clinicians with respect to therapeutic recommendations, treatment expectations and severe complications that may occur during the evolution of a disease. Despite RDRs comprising small patient numbers, the burden of RDs on patients, families, health systems and the economy is proportionally higher than the general population [20]. Therefore, having a coordinated approach to collecting RD data may provide economies of scale and thereby enable a wider positive impact on patient outcomes [5].

RDs can lead to a significant reduction in quality of life for patients and their families. Ensuring the patient voice is central to clinical decision making is key to delivering, evaluating and understanding the efficacy of therapeutic interventions [21]. PROMs are highly effective for ensuring the patient voice informs best practice care [22, 23]. The collection of PROMs by RDRs offers the potential to improve patient care and clinical outcomes [21, 24]. Nonetheless, only 40% RDRs reported capturing PROMs in our study. One reason for this may be that administering PROMs for RDs poses unique challenges, including small patient populations, disease heterogeneity and a lack of natural history knowledge [25]. Given the small number of people living with individual RDs, PROMs should be supported by RDRs [21].

Similar surveys have been conducted in other countries. In 2011, the European Commission funded the EPIRARE project (‘Building Consensus and Synergies for the European Registration of Rare Disease Patients’), to inform the development of a European Platform for RDRs [26–28]. The EPIRARE survey investigated the minimum data elements of RDRs, to address various methodological, technical, and regulatory issues, and ways to find resources to develop and sustain registries. Of the 272 registries surveyed, 48% did not have a clear strategy for long-term sustainability, 34% did not have a specific management group, 30% did not share data, and 21% were established without any clear funding [26]. The results of the survey demonstrated various issues regarding financial support, data quality, and the need to improve data sharing. Moreover, the registry holders were supportive of a common platform for RD registries.

Ali et al. [15] conducted an international survey of RDR leaders to ascertain level of consensus regarding the quality criteria that should be considered essential features of a RDR. Of 35 respondents representing 40 RDRs, over 95% said that essential quality criteria should include establishment of a good governance system (ethics approval, registry management team, standard operating protocol and long-term sustainability plan), data quality (personnel responsible for data entry and procedures for checking data quality) and construction of an IT infrastructure.

A survey of RDRs in Japan reported similar challenges to RDR’s surveyed as part of this Australian study, including difficulties securing sustainable funding, reducing operational burdens, gaining cooperation from participants and promoting the use of data for research or to improve health outcomes [29]. To address these issues, the Japanese study proposed to: (1) build a common platform with universal facilities, (2) establish collaboration with educational institutions to increase awareness of RDRs, and (3) secure a sufficient budget from collaboration between academia and industry to maintain RDRs.

Our findings are similar to those reported above, supporting the need for a nationally coordinated approach of RD data capture. To achieve this in Australia, well-coordinated efforts should involve all stakeholders [5]. Dedicated funding and incentives should be allocated for RDRs to ensure full coverage of the eligible patient population. Investing in the infrastructure and staffing would assist in streamlining and simplifying the maintenance and data entry demands of RDRs across Australia. Finally, RDRs should be promoted in hospitals and relevant clinics, and collaborations with relevant international registries should be sought [11].

### Strengths and limitations

Integration of the quantitative and qualitative study components has strengthened the validity of the findings and provided a richer understanding of participants’ experiences regarding the RVA strategy towards building a national RDR in Australia. A major limitation of our study was that of the 191 survey recipients only a small number participated in the survey and qualitative interviews despite invitations and reminders. Reasons for the low rate of participation are unfortunately unknown, although small staffing of RDRs may be associated. Furthermore, this was a cross-sectional study, therefore responses provided could only give a snapshot experience at a given time.

## Conclusions

The results of our study highlighted that while RDRs are feasible and valuable to multiple stakeholders, there are key principles required for success. These include developing a process of low burden data collection; having sustainable funding; contributions of consumer wellbeing information; and ensuring multiple data uses, including reporting, epidemiology, secondary research and access to clinical trials. Opportunities to consolidate existing RDR information and ensure access to RDR expertise to support new RDRs is a starting point to realising the potential of RDRs in Australia. Precision medicine is likely to see more RDRs established to support clinical trials and post-marketing surveillance. Having a co-ordinated approach nationally will enable maximum impact from RDRs in Australia, and maximum benefit for people with RDs.

### Electronic supplementary material

Below is the link to the electronic supplementary material.

Supplementary Material 1

## Data Availability

The datasets used and/or analysed during the current study are available from the corresponding author on reasonable request.

## Abbreviations

ACFDR: Australian Cystic Fibrosis Data Registry
AILDR: Australasian Interstitial Lung Disease Registry
AIPFR: Australian Idiopathic Pulmonary Fibrosis Registry
aHUS: Atypical Haemolytic Uremic Syndrome
ANCJDR: Australian National Creutzfeldt-Jakob Disease Registry
HbR: Haemoglobinopathy Registry
LAL-D: Lysosomal Acid Lipase Deficiency
MRDR: Myeloma and Related Diseases Registry
NAIT: Neonatal Alloimmune Thrombocytopenia
PROMs: Patient Reported Outcome Measures
RD: Rare Disease
RVA: Rare Voices Australia
RDRs: Rare Disease Registries
SMAC: Scientific and Medical Advisory Committee
