# Supplementary material for: Informing a national rare disease registry strategy in Australia: a mixed methods study
Source: BMC Health Serv Res. 2023 Oct 31;23:1187. doi: 10.1186/s12913-023-10049-x (PMC10619239; doi:10.1186/s12913-023-10049-x)
Supplement: Supplementary file 1 — Supplementary Material 1 [file 12913_2023_10049_MOESM1_ESM.docx]

**Topic guide**

Thank you for agreeing to be interviewed. Can I double check that you have read the Information and Consent Form? Do you have any questions before I begin?

As you are aware the purpose of this project is:

1. To help us learn from your knowledge and experience and to gather your views on the success your registry has been able to achieve;
2. To better understand the resources required for your registry;
3. Understand, the coverage, attributes and feedback mechanisms to the different; stakeholders: clinicians, managers, policymakers and researchers;
4. Understand the barriers and enablers in achieving the goals of the registry.

For the sake of accuracy, I’d like to record our conversation. Is that ok with you? I’ll put the phone on speaker. I want to assure you that everything we talk about will be confidential. If we write up our research, all your details will be de-identified.

Is there anything you’d like to ask me before we begin?

**Questions:**

1. What are its objectives and research priorities?
2. To what extent did you investigate what others were doing before setting up the registry?
3. How is your registry funded (e.g. through industry, government, donations)?
4. With the rise in shared decision-making, individuals and community organisations are increasingly regarded as research partners. Is there patient/consumer representation on your steering committee? To what extent? Please elaborate.
5. Post-marketing surveillance is the practice of monitoring the safety of a pharmaceutical drug or medical device after it has been released on the market. Has your registry data been utilised for post-marketing surveillance for high-host medicines and what impact has it made?
6. Overall, to what extent are you aware that your registry has made an impact? To what extent has the impact been quantified?
7. Can you please describe the main challenges you face in meeting the objectives of your registry? What strategies have you adopted to address these challenges and how effective they were?
8. Rare Voices Australia (RVA) is the national peak body for Australians living with a rare disease. RVA has been calling for a nationally coordinated approach to rare disease registries. A key action is to *‘develop a national approach to person-centred rare disease registries to support national standards, best practice and minimum data sets*.’ Would you support this action, and how would you feel about a national rare disease registry? Please elaborate.
